# ActivinA modulates B-acute lymphoblastic leukaemia cell communication and survival by inducing extracellular vesicles production

**DOI:** 10.1038/s41598-024-66779-3

**Published:** 2024-07-12

**Authors:** Eugenia Licari, Giulia Cricrì, Mario Mauri, Francesca Raimondo, Laura Dioni, Chiara Favero, Alice Giussani, Rita Starace, Silvia Nucera, Andrea Biondi, Rocco Piazza, Valentina Bollati, Erica Dander, Giovanna D’Amico

**Affiliations:** 1 Tettamanti Center, Fondazione IRCCS San Gerardo dei Tintori, Via Pergolesi, 20900 Monza, Italy; 2 Paediatric Nephrology, Dialysis and Transplant Unit, Fondazione Ca’ Granda IRCCS Ospedale Maggiore Policlinico, Milano, Italy; 3 Department of Medicine and Surgery, University of Milano-Bicocca, Monza, Italy; 4 Clinical Proteomics and Metabolomic Unit, School of Medicine and Surgery, University of Milano-Bicocca, Monza, Italy; 5 EPIGET Lab, Department of Clinical Sciences and Community Health, University of Milan, Milan, Italy; 6 Pediatrics, Fondazione IRCCS San Gerardo dei Tintori, Monza, Italy; 7 Hematology Division and Bone Marrow Unit, Fondazione IRCCS San Gerardo dei Tintori, Monza, Italy; 8 CRC, Center for Environmental Health, University of Milan, Milan, Italy; 9 Occupational Health Unit, Fondazione IRCCS Ca’ Granda-Ospedale Maggiore Policlinico, Milan, Italy

**Keywords:** B-cell acute lymphoblastic leukaemia, ActivinA, Microenvironment, Extracellular vesicles, Intercellular communication, Cell survival, Cancer microenvironment, Cell death

## Abstract

Extracellular vesicles (EVs) are a new mechanism of cellular communication, by delivering their cargo into target cells to modulate molecular pathways. EV-mediated crosstalk contributes to tumor survival and resistance to cellular stress. However, the role of EVs in B-cell Acute Lymphoblastic Leukaemia (B-ALL) awaits to be thoroughly investigated. We recently published that ActivinA increases intracellular calcium levels and promotes actin polymerization in B-ALL cells. These biological processes guide cytoskeleton reorganization, which is a crucial event for EV secretion and internalization. Hence, we investigated the role of EVs in the context of B-ALL and the impact of ActivinA on this phenomenon. We demonstrated that leukemic cells release a higher number of EVs in response to ActivinA treatment, and they can actively uptake EVs released by other B-ALL cells. Under culture-induced stress conditions, EVs coculture promoted cell survival in B-ALL cells in a dose-dependent manner. Direct stimulation of B-ALL cells with ActivinA or with EVs isolated from ActivinA-stimulated cells was even more effective in preventing cell death. This effect can be possibly ascribed to the increase of vesiculation and modifications of EV-associated microRNAs induced by ActivinA. These data demonstrate that ActivinA boosts EV-mediated B-ALL crosstalk, improving leukemia survival in stress conditions.

## Introduction

Among the several types of the acute lymphoblastic leukaemia (ALL) malignancies, B-cell ALL (B-ALL) represents the majority of ALL cases in children^1^*.* Although the current therapeutic regimens improved long-term survival rate of B-ALL to more than 85%, after complete remission, around the 20% of the cases relapse and became refractory^1^*.* An important contribution to treatment failure has been ascribed to the protective role of the altered leukemic bone marrow (BM) microenvironment, a sanctuary in which stromal cells communicate with leukemic B cells by taking direct contact, secreting soluble factors, including metabolites and releasing extracellular vesicles (EVs)^2^ ^3^. In order to improve disease outcome of B-ALL patients, the identification and the targeting of new altered molecules involved in the leukemia-stroma crosstalk could be used in combination with conventional chemotherapy. With this purpose, we focused on ActivinA, a cytokine belonging to the TGF-β family, that we found upregulated within the B-ALL BM compared to the BM of healthy donors. Moreover, we discovered that ActivinA is specifically produced and secreted by mesenchymal stromal cells (MSCs) after culture with leukemic cells^4^. Several studies have identified ActivinA as an important regulator of cancer progression in solid tumors^5,6^. In the context of B-ALL, we demonstrated that ActivinA exerts a pro-leukemic action by increasing the migratory and invasive properties of leukemic cells, by promoting the intracellular calcium flux and actin polymerization^4^. Similarly to cell migration, also EV release is dependent upon cytoskeleton activation and membrane remodeling^7^. As carriers of several functional molecules, especially a group of small non-coding RNA named microRNA (miRNA), EVs are considered a new important way of intercellular communication^8,9^. EVs have been deeply investigated in the pathogenesis of solid tumors, and increasing evidence reports that cancer cells can exchange EVs to improve their survival and therapy resistance. In this context, miRNA transferred by EVs can downregulate specific mRNA target in the recipient cancer cells, modulating relevant biological/pathological processes^10^. In breast cancer, EVs from MCF-7 cells resistant to tamoxifen have been shown to transfer miR-221/222 to other breast cancer cells enhancing tamoxifen-resistance^11^*.* In addition, in pancreatic cancer the miRNA transported by EVs have been linked to inhibition of cell apoptosis. Indeed, exosomes from pancreatic cancer cells can promote cancer cell growth and chemotherapy resistance by transferring miR-155^12^. On the contrary, few papers have described the role of EVs in leukemia pathogenesis. In this context, it has been reported that EVs from chemoresistant promyelocytic leukemia HL60 cells can transfer their chemoresistance potential to drug sensitive HL60 cells by delivering miRNAs, nucleic acids and proteins^13^*.* Moreover, in chronic lymphocytic leukemia (CLL), EVs have been demonstrated to play a key role in the crosstalk between B-cells and the BM microenvironment. Accordingly, EVs isolated by BM-MSCs decreased B-CLL cell apoptosis, while increasing their resistance to different drugs^14^. Overall, the functions of EVs in B-ALL remain so far to be elucidated. In the current study, we investigated for the first time the effect of ActivinA on B-ALL cell vesiculation and the potential impact on leukemic cell survival under restrictive conditions, such as nutrient starvation, typically observed in a highly infiltrated leukemic BM.

## Results

### B-ALL cells release EVs in vitro expressing comparable markers independently of ActivinA stimulation

**Figure 1:**
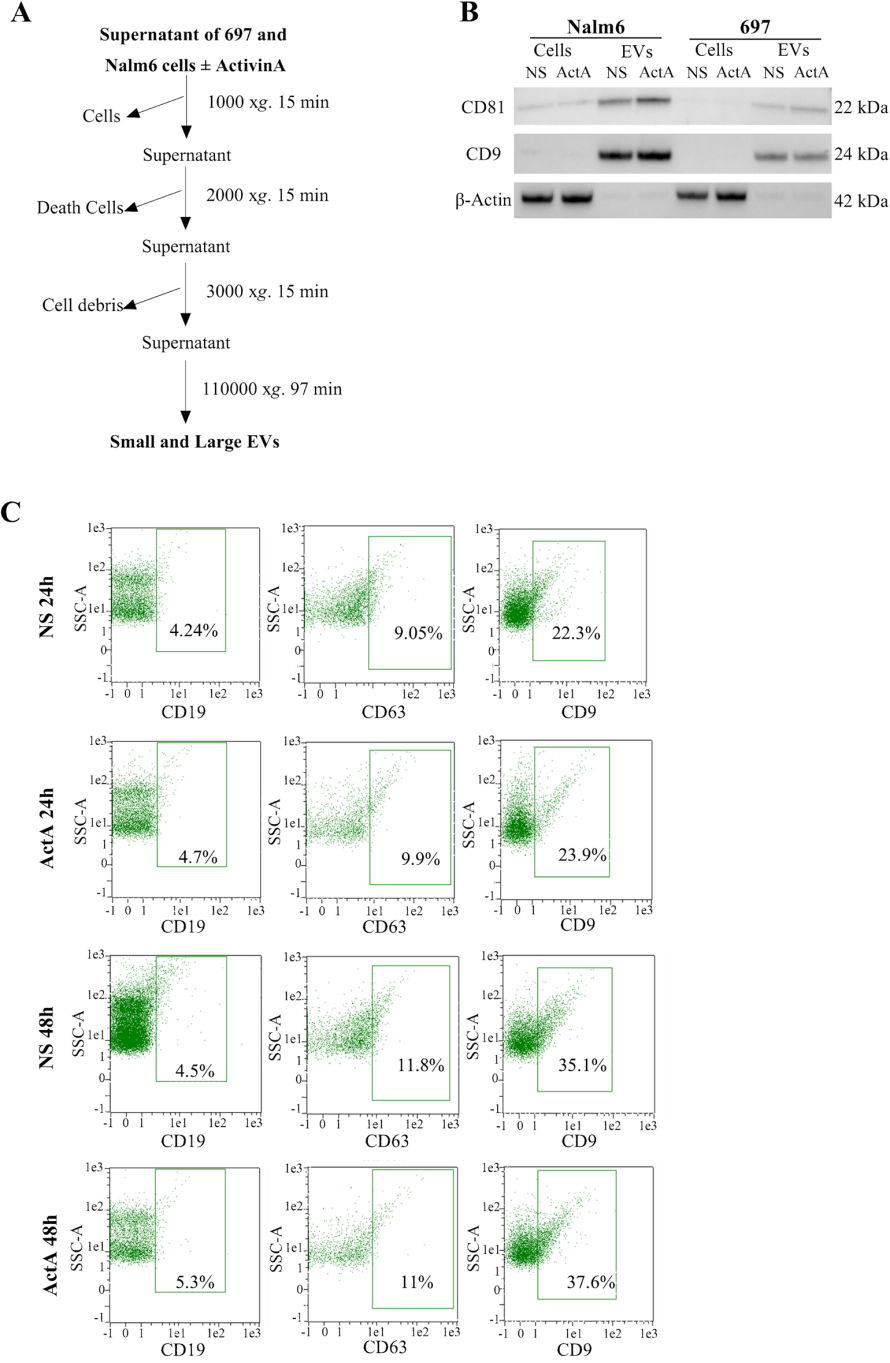
Characterization of EVs. **(A)** Isolation protocol for EVs. After 24 or 48 h of culture in serum-free medium, cell supernatants were centrifuged three times with increasing speed. Then, the samples were ultracentrifuged at 110,000×*g* for 97 min to pellet total EVs. **(B)** Enrichment of EV marker. Western blot analysis of CD81 and CD9 tetraspanins and β-actin on 697 and Nalm6 cell lysates (cells) and EVs isolated after 24 h of culture in serum-free medium alone (NS) or in presence of ActivinA (ActA). The blots are representative of two independent experiments. **(C)** Flow cytometry evaluation of CD9 and CD63 tetraspanins and B-cell marker CD19 on EVs isolated from 697 cells stimulated or not with ActivinA for 24 or 48 h. The plots are representative of three independent experiments (CD19/CD63) and two independent experiments for CD9.

In order to investigate the impact of ActivinA on B-ALL vesiculation, we took advantage of two B-ALL cell lines: 697 t(1;19) and Nalm6 t(5;12). We cultured 697 and Nalm6 cells at the concentration of 2 × 10^6^ cell/ml in serum-free medium to avoid contaminating aggregates interfering with the following analysis. Hence, we firstly investigated the effect of serum deprivation on B-ALL cell lines proliferation. Even if the growth of the 697 and Nalm6 cells in FBS-free conditions significantly decreased compared to the complete medium condition (10% FBS), the cells were phenotypically normal at microscope and viable (Supplementary Fig. 1). Consequently, 40 × 10^6^ cells were seeded in serum-free RPMI 1640 and stimulated or not with ActivinA 50 ng/ml or 200 ng/ml for 24 and 48 h. For all the experimental conditions the supernatants were analyzed by NTA to evaluate the concentration of particle/ml and the mean EV size, or used to purify EVs to test their identity by evaluating the expression of typical EV markers. To isolate EVs we exploited an optimized protocol which consists of three differential centrifugation steps and one round of ultracentrifugation, as previously described in literature and illustrated in Fig. 1A^15^. To verify the identity of isolated EVs, the enrichment of the two tetraspanins CD81 and CD9 was evaluated by western blot. As expected, both CD81 and CD9 were enriched in EVs isolated from both 697 and Nalm6 cell lines, whereas they were faintly expressed in the correspondent cells (Fig. 1B). On the contrary, β-Actin resulted well-expressed in the cell, but almost absent in the EVs, as previously demonstrated^16^. Importantly, ActivinA stimulation did not impact on CD81 and CD9 expression, neither in the cells nor in the EVs (Fig. 1B). Alongside, we performed an immunophenotypical analysis of 697-derived EVs by flow cytometry. To confirm EV identity, we evaluated the expression of CD19, which is a typical B cell-marker. CD19 was expressed on EVs isolated from both 697 cells stimulated or not with ActivinA (EV-NS and EV-ActA respectively), at comparable levels between the two groups at 24 h (EV-NS: mean  4.24%, range  3.82–6.03%, EV-ActA: mean  4.7%, range  3.83–7.74%) as well as at 48 h (EV-NS: mean 4.5%, range 4.45–7.91%, EV-ActA: mean  5.3%, range  4.93–7.71%, n = 3). Moreover, we revealed the presence of tetraspanin CD63, another EV protein marker, on both EV-NS (24 h: mean  9.05%, range  4.9–9.72%, 48 h: mean  11.8%, range  7.36–11.69%, respectively) and EV-ActA (24 h: mean  9.9%, range  6.26–11.96%, 48 h: mean  11%, range  8.43–11.01%, n = 3) (Fig. 1C). CD9 was the most expressed tetraspanin in EV-NS (24 h: mean  22.3%, range  21.78–24%, 48 h: mean  35.1%, range  31.22–37.41%, respectively) and EV-ActA (24 h: mean  23.9%, range 21.96–26.75%, 48 h: mean  37.6%, range  31.57–39.75%, n = 2).

### ActivinA increases B-ALL cell vesiculation

**Figure 2:**
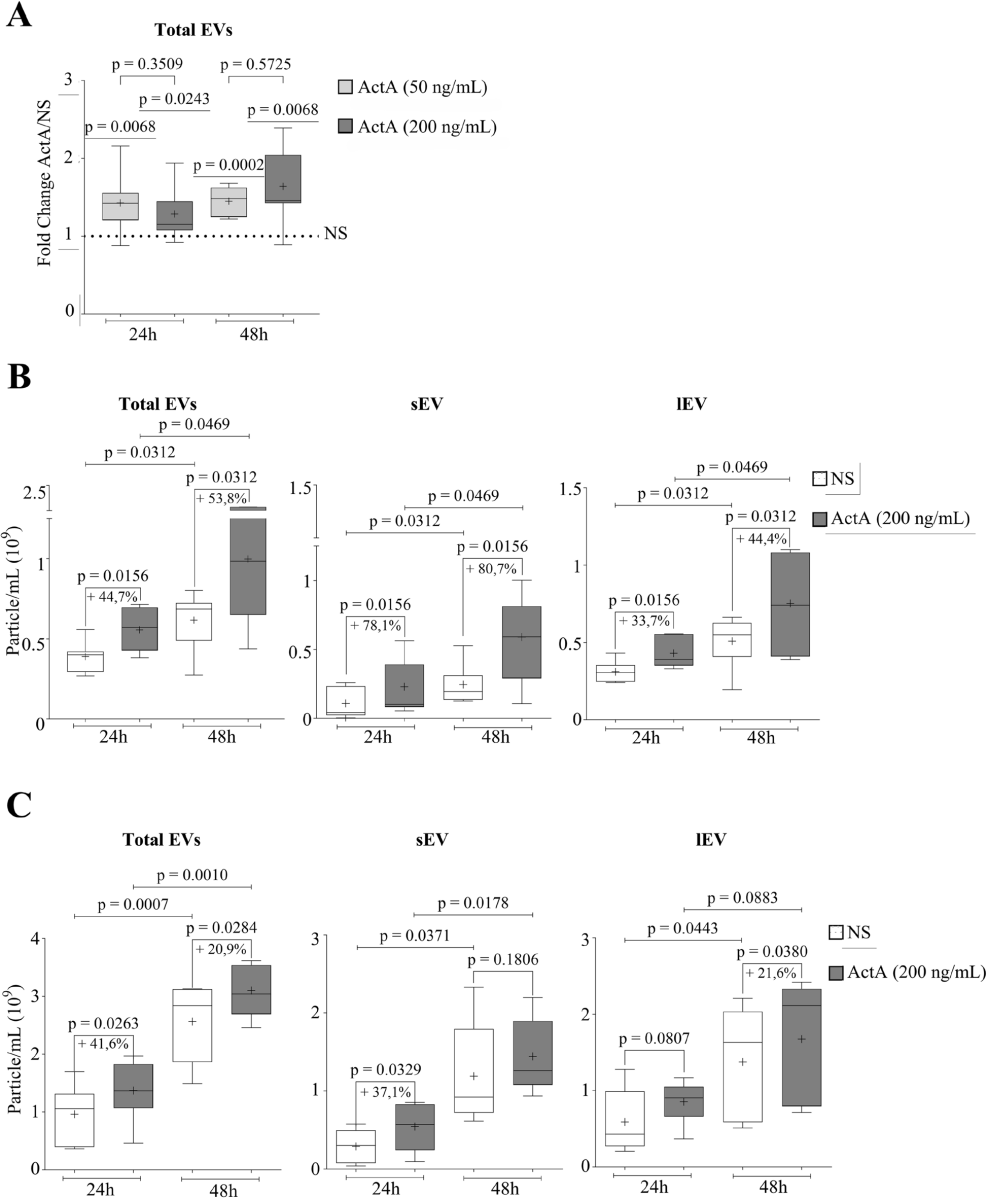
ActivinA upregulates EV release from B-ALL cells. **(A)** 697 cells were pretreated or not with ActivinA 50 ng/ml or 200 ng/ml, for 24 and 48 h. The concentration (particle/ml) of EVs in the supernatant was determined by means of NTA. Fold change (FC) was calculated as number of particle/ml in the supernatant of ActivinA-stimulated cells (ActA)/not stimulated (NS) cells. Each box plot shows the median, the mean (+) and extends from the lowest to the highest value (n ≥ 8 independent experiments per condition). Two-tailed one sample t test: FC ActA/NS vs 1; Mann–Whitney two-tailed test: ActivinA 200 ng/ml vs ActivinA 50 ng/ml. **(B,C)** Box plot graphs representing the concentration of total EVs, sEV and lEV released by 697 (**B**) or Nalm6 cells (**C**) pretreated or not with ActivinA 200 ng/ml for 24 and 48 h. Each box plot shows the median, the mean (+) and extends from the lowest to the highest value (*n* = 7 for 697 cells or n ≥ 5 for Nalm6 cells independent experiments per condition). Wilcoxon matched-pairs two-tailed test.

To investigate whether ActivinA could influence EV production by B-ALL cells, we performed NTA analysis on 697 cell supernatants after 24 or 48 h of culture in serum-free medium in presence or not of ActivinA at two different doses (50 ng/ml and 200 ng/ml). As shown in Fig. 2A, ActivinA was able to significantly increase the number of total EVs at all the time points tested, with no differences between the two doses. In detail, 200 ng/ml of ActivinA increased the total number of EVs of 44.7% at 24 h and 53.8% at 48 h compared to the NS condition (Fig. 2B). We next analyzed EV production by dividing small EVs (sEV) from large EVs (lEV) based on their size (size range: 30–150 nm and 151–750 nm, respectively). After 24 h of treatment, 200 ng/ml ActivinA increased the production of sEV of 78.1% (sEV-ActA mean concentration (MC):1.46E + 08 particle/mL, range 2.16E + 07–2.25E + 08 particle/mL), compared to NS cells (sEV-NS MC: 8.21E + 07 particle/mL, range:1.71E + 06–1.04E + 008 particle/mL, p < 0.01, n = 8). Similarly, lEV were increased of 33.7% (lEV-ActA MC: 4.07E + 08 particle/mL, range: 2.4E + 08–5.56E + 08 particle/mL), compared to NS cells (lEV-NS MC: 3.04E + 08 particle/mL, range:2.09E + 08–4.31E + 08 particle/mL, p < 0.01, n = 8) (Fig. 2B). At 48 h, sEV production by ActivinA-stimulated 697 cells was 80.7% higher (MC: 2.92E + 08 particle/mL, range: 1.01E + 07–4.01E + 08 particle/mL) than the NS counterpart (MC: 1.62E + 08 particle/mL, range: 5.08E + 07–2.11E + 08 particle/mL, p < 0.03, n = 8), and also the lEV released in response to ActivinA was increased of about 44.4% (MC: 6.67E + 08 particle/mL, range: 1.05E + 08–1.10E + 09 particle/mL), compared to the basal condition (MC: 4.62E + 08 particle/mL, range 8.01E + 07–6.63E + 08 particle/mL, p < 0.03, n = 8) (Fig. 2B). These results indicate a clear effect of ActivinA on B-ALL cell vesiculation. Moreover, as illustrated in Fig. 2B, the quantity of sEV and lEV increased time dependently under basal conditions (NS 48 h vs NS 24 h), as well as upon ActivinA stimulation (ActA 48 h vs ActA 24 h). A significant increment of both EV subpopulations was also observed when using 50 ng/ml ActivinA compared to NS cells, as illustrated in Supplementary Fig. 2. The ability of ActivinA to induce B-ALL cell vesiculation was also confirmed on the Nalm6 cell line (Fig. 2C). Indeed, similarly to 697 cells, the total quantity of EVs released by Nalm6 cells was significantly increased by ActivinA (200 ng/ml) both at 24 and 48 h, with sEV showing the highest increase at 24 h and lEV at 48 h (Fig. 2C). ActivinA stimulation (200 ng/ml) was able to increase at 24 h the release of total EVs, especially promoting lEVs, also in another B-ALL cell line, namely the SUPB-15, bearing the t(9;22) (Supplementary Fig. 3). These data suggest that ActivinA promotion of B-ALL vesiculation could be karyotype independent. Concerning EV size, their mean was not modulated by ActivinA (200 ng/ml) at 24 and 48 h, both in case of 697 and Nalm6 cells (Supplementary Fig. 4A, B). However, EV mean size decreased in a time-dependent manner in 697 cells unstimulated or stimulated with ActivinA (Supplementary Fig. 4A). This reduction in EV diameter (nm) was not instead observed in the Nalm6 cell line (Supplementary Fig. 4B).

### ActivinA promotes vesicle-mediated crosstalk between B-ALL cells

**Figure 3:**
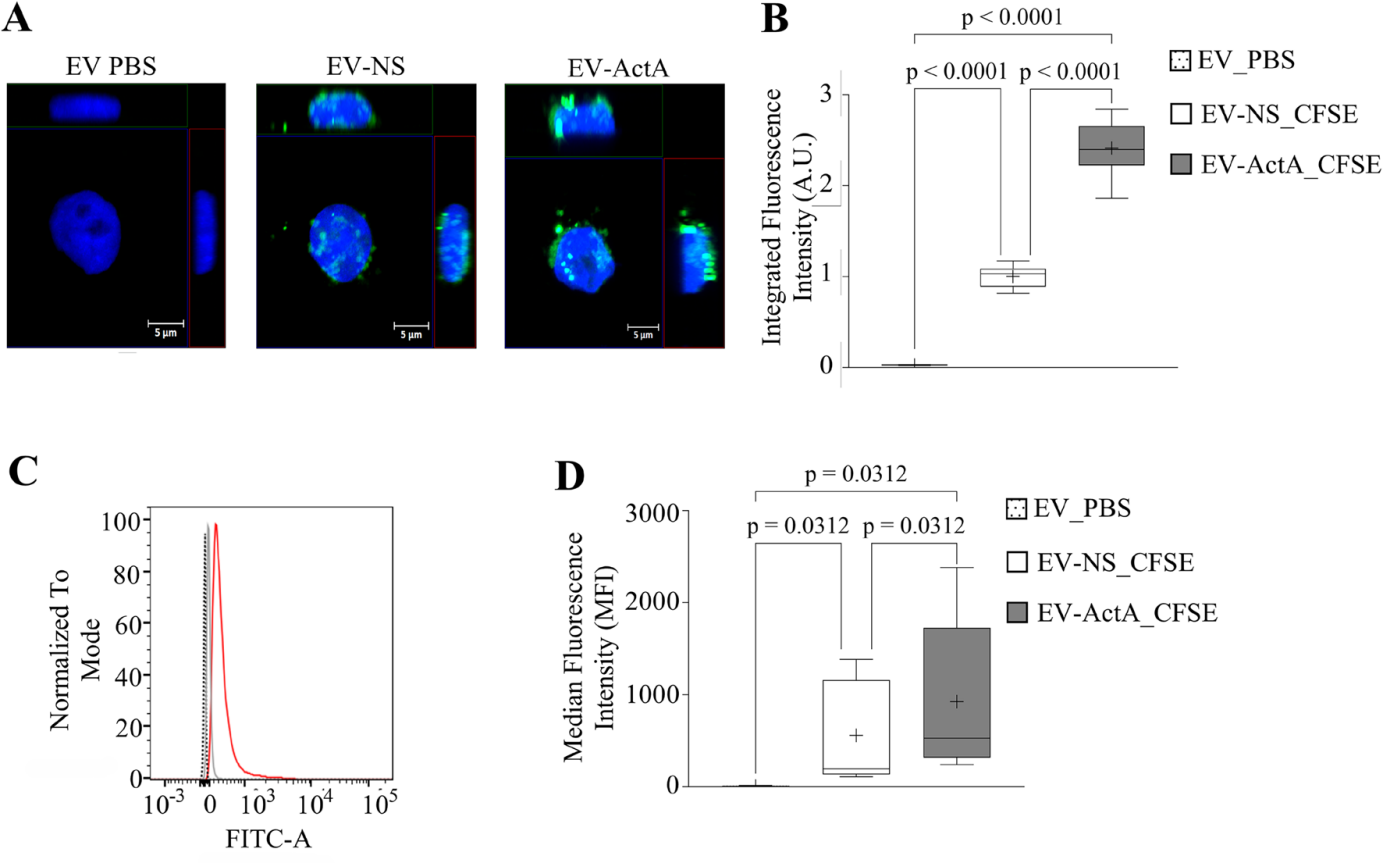
B-ALL cells can crosstalk through the internalization of released EVs. EVs were isolated from 697 cells stimulated (EV-ActA) or not (EV-NS) with ActivinA (200 ng/ml). EV-NS and EV-ActA were stained with CFSE (green fluorescent dye) or PBS (EV-PBS, negative control) and cocultured with 697 cells for 24 h. **(A)** Representative confocal 3D maximum intensity reconstruction of 697 cells after internalization of EV-PBS, EV-NS and EV-ActA. The green fluorescent dots around the cell nucleus (DAPI staining, blue) indicate internalized EVs. **(B)** Confocal microscopy quantification of CFSE Integrated Fluorescence Intensity. Each box plot shows the median and the mean (+) and extends from the lowest to the highest value (*n* = 3 independent experiments). Two-way ANOVA with Tukey’s correction for multiple comparisons. **(C)** Representative overlay histogram showing CFSE fluorescence evaluated by flow cytometry in 697 cells cultured with CFSE-stained EV-NS (gray) or EV-ActA (red). Unstained 697 cells were used as negative control (dashed gray line). **(D)** Flow cytometry quantification of CFSE MFI in 697 cells cultured with CFSE-labeled EV-NS and EV-ActA. Each box plot shows the median, the mean (+) and extends from the lowest to the highest value (*n* = 5 independent experiments). Wilcoxon matched-pairs two-tailed test.

So far, we demonstrated that B-ALL cells are able to release EVs and ActivinA treatment boosts EV production. To understand whether ActivinA, by increasing the quantity of EVs, could impact on the crosstalk between B-ALL cells, we further investigated EV uptake. With this purpose, a comparable amount of 697 cells was stimulated or not with ActivinA and, 24 h-supernatants were collected to isolate total EVs (as previously described). The obtained EVs were stained with CFSE or not (EV PBS = negative control) and added to 697 cells cultured under standard conditions. Hence, the ability of recipient 697 cells to internalize EVs was assessed in parallel by confocal microscopy (Fig. 3A, B) and flow cytometry (Fig. 3C, D). As shown in Fig. 3A, confocal microscopy clearly revealed that 697 cells were able to uptake EVs isolated from other leukemic cells stimulated (EV-ActA) or not (EV-NS) with ActivinA and visualized as green dots around the cell nucleus (DAPI staining, blue). Interestingly, Integrated Fluorescence Intensity was higher when 697 cells were cocultured with EV-ActA (mean Arbitrary Units (A.U): 2.41, range 1.86–2.84), compared to EV-NS (mean A.U.: 1, range 0.85–1.17, n = 3) (Fig. 3B). These data suggest that 697 cells receiving EV-ActA internalized an almost doubled quantity of EVs. Remarkably, also the flow cytometry quantification of the CFSE signal, after coculture of leukemic cells with EVs, showed a higher fluorescence signal in 697 cells that received EV-ActA, compared to 697 cells that received EV-NS (Fig. 3C, D). In detail, the CFS Median Fluorescence Intensity (MFI) was 924 (range 240–2381) for 697 cells cocultured with EV-ActA, while the MFI was 555 in the case of EV-NS (range 106–1386, p = 0.002, n = 5) (Fig. 3D). Comparable results were obtained by using the Nalm6 cell line (Supplementary Fig. 5). Overall, these data suggest that ActivinA, by stimulating B-ALL cell vesiculation, could also increase the exchange of information with other leukemic cells through the internalization of vesicle cargo.

### EV stimulation promotes the survival of 697 B-ALL cells in vitro cultured under stress conditions in a dose-dependent manner

**Figure 4:**
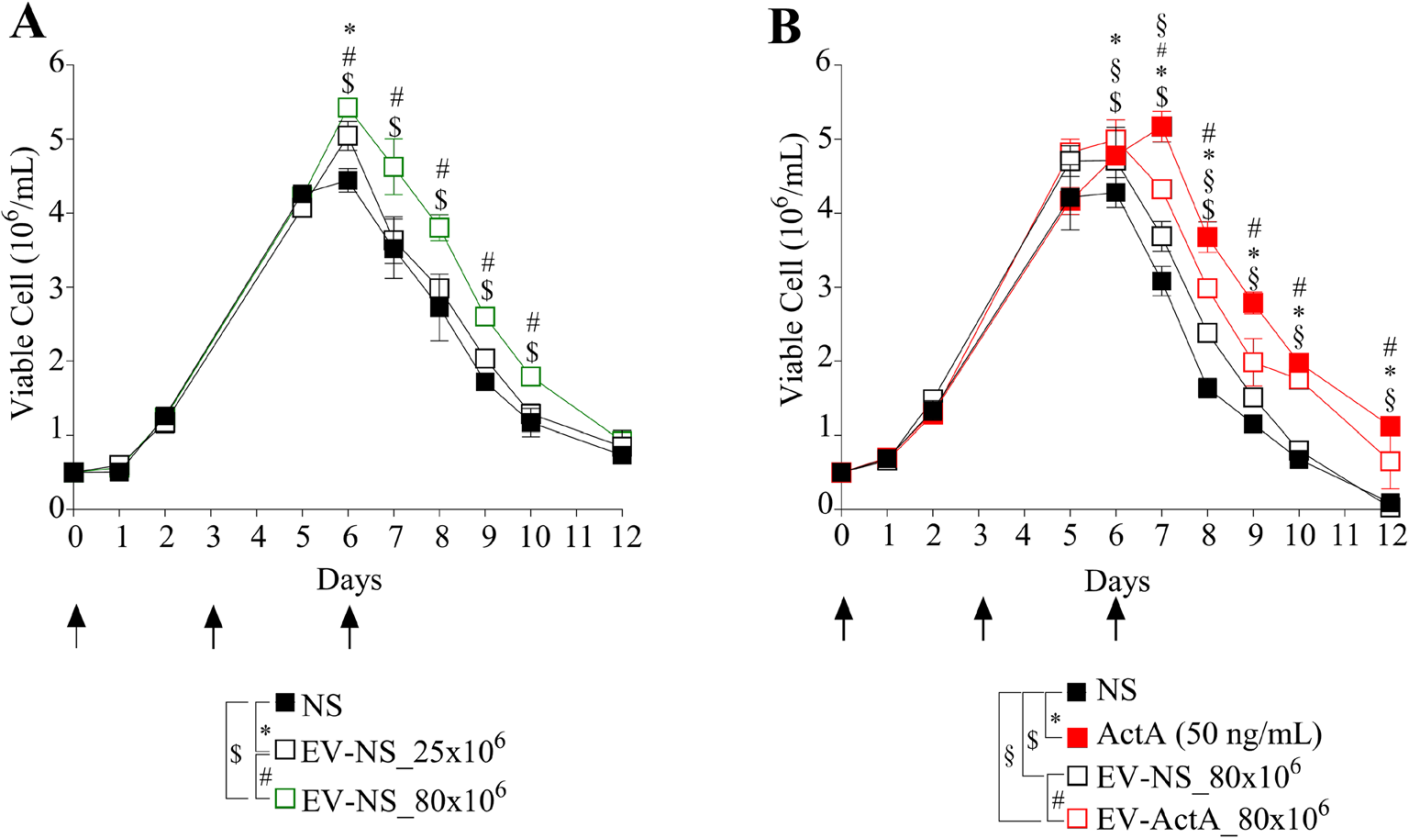
B-ALL cell crosstalk mediated by EV release contributes to cell viability modulation. **(A)** EV-NS were isolated from the supernatant of 25 × 10^6^ (EV-NS_25 × 10^6^, black empty square) or 80 × 10^6^ (EV-NS_80 × 10^6^, green empty square) unstimulated 697 cells and added on day 0, 3 and 6 (black arrows) to 697 cells that were kept in culture for 12 days without changing medium. PBS-stimulated 697 cells were used as unstimulated control (NS, black square). The panel shows the growth curves obtained by counting the number of viable cells by an automated cell counter in three wells for each condition (mean ± sd). One representative experiment out of three independent cultures performed is shown. *p: NS vs EV-NS_25 × 10^6^, ^#^p: EV-NS_25 × 10^6^ vs EV-NS_80 × 10^6^, ^$^p: NS vs EV-NS_80 × 10^6^, two-way ANOVA with Tukey’s correction for multiple comparison, all the actual p values are reported in Supplementary Table 1. **(B)** 697 cells were stimulated or not with ActivinA 200 ng/ml (red square) or with PBS (NS, black square) on day 0, 3 and 6 (black arrows). EVs were isolated from the supernatant of 80 × 10^6^ 697 cells unstimulated (EV-NS_80 × 10^6^, black empty square) or stimulated with ActivinA (EV-ActA_80 × 10^6^, red empty square) and added on day 0, 3 and 6 (black arrows) to 697 cells that were kept in culture for 12 days without changing medium. The panel shows the growth curves obtained by counting the number of viable cells by an automated cell counter in three wells for each condition (mean ± sd). One representative experiment out of three independent cultures performed is shown. *p: NS vs ActA (50 ng/mL), ^$^p: NS vs EV-NS_80 × 10^6^, ^#^p: EV-NS_80 × 10^6^ vs EV-ActA_80 × 10^6^, ^§^p: NS vs EV-ActA_80 × 10^6^, two-way ANOVA with Tukey’s correction for multiple comparison, all the actual p values are reported in Supplementary Table 1.

It has been previously demonstrated that EVs can modulate apoptosis in diverse tumors, including some types of leukemia^11–14^*.* Few evidence indicates that exosomes can modulate cell survival also in the context of B-ALL^17^. Therefore, we investigated more in depth whether B-ALL-derived EVs could exert a pro-survival effect on neighboring B-ALL cells. With this aim, 697 cells were cultured for 12 days without changing their growth medium, treated three times on day 0, 3 and 6 with two different doses of EV-NS (Fig. 4A, black arrows) and evaluated in terms of number of viable cells. As shown in Fig. 4A, in the exponential growth phase we did not observe any significant difference among 697 cells treated with EV-NS isolated from 25 × 10^6^ or 80 × 10^6^ 697 cells and vehicle-treated 697 cells (NS), suggesting that EV uptake does not impact cell proliferation. On the contrary, at plateau (day 6), when NS cells drastically started to die, probably because of the lack of nutrients, 697 cells treated with EV-NS showed a significant viability advantage. Specifically, 697 cells treated with the lower dose of EV-NS showed a modest increase of viable cells compared to control, which was significant only at day 6 (fold increase = 15%, p = 0.02). Interestingly, under stress conditions, the higher EV-NS dose (80 × 10^6^) exhibited a more pronounced ability to protect leukemic cells from death as until day 10, the number of viable cells was about 50% higher compared to NS 697 cells (Fig. 4A). Importantly, at day 7, 8 and 9, the 697 cells which received the EV-NS 80 × 10^6^ showed a 28% higher number of viable cells than 697 cells treated with EV-NS 25 × 10^6^ (Fig. 4A). These findings demonstrate that the EV-NS exert a pro-survival effect on 697 cells in a dose-dependent manner.

Taking into consideration that ActivinA boosts B-ALL cell vesiculation, we tested the efficacy of ActivinA and EV-ACTA to promote cell survival using the above-described experimental setting. The graph illustrated in Fig. 4B shows that ActivinA stimulation on days 0, 3 and 6 strongly impacted on 697 cell survival from day 6 up to day 12. In detail, at day 7 of the growth curve, the number of viable 697 cells was 67% higher in case of ActivinA stimulation, compared to the NS condition. The following days, the number of viable cells was almost doubled in the ActivinA condition compared to NS (Fig. 4B), demonstrating for the first time that ActivinA can directly promote B-ALL cell survival under stress conditions. Interestingly, EV-ActA showed an increased ability to protect 697 cells from cell death than EV-NS isolated from the same number of 697 cells. The difference between EV-ActA and EV-NS on cell survival was statistically significant starting from day 7 in which the amount of viable 697 cells treated with EV-ActA was increased of 17% compared to 697 cells which received EV-NS. Of note, the protective capacity of EV-ActA became more evident in the following days. The quantity of viable cells, at day 8 and 9, was respectively 25% and 31% higher in the EV-ActA condition, whereas at day 10, viable cells were doubled in the EV-ActA group compared to the EV-NS. Interestingly, ActivinA promoted 697 cell survival at a higher extent compared to EV-ActA, suggesting that the increase of B-ALL cell vesiculation could be one of the pro-survival mechanisms exerted by this cytokine.

### ActivinA modulates the miRNA cargo of EV released by 697 cells

**Figure 5:**
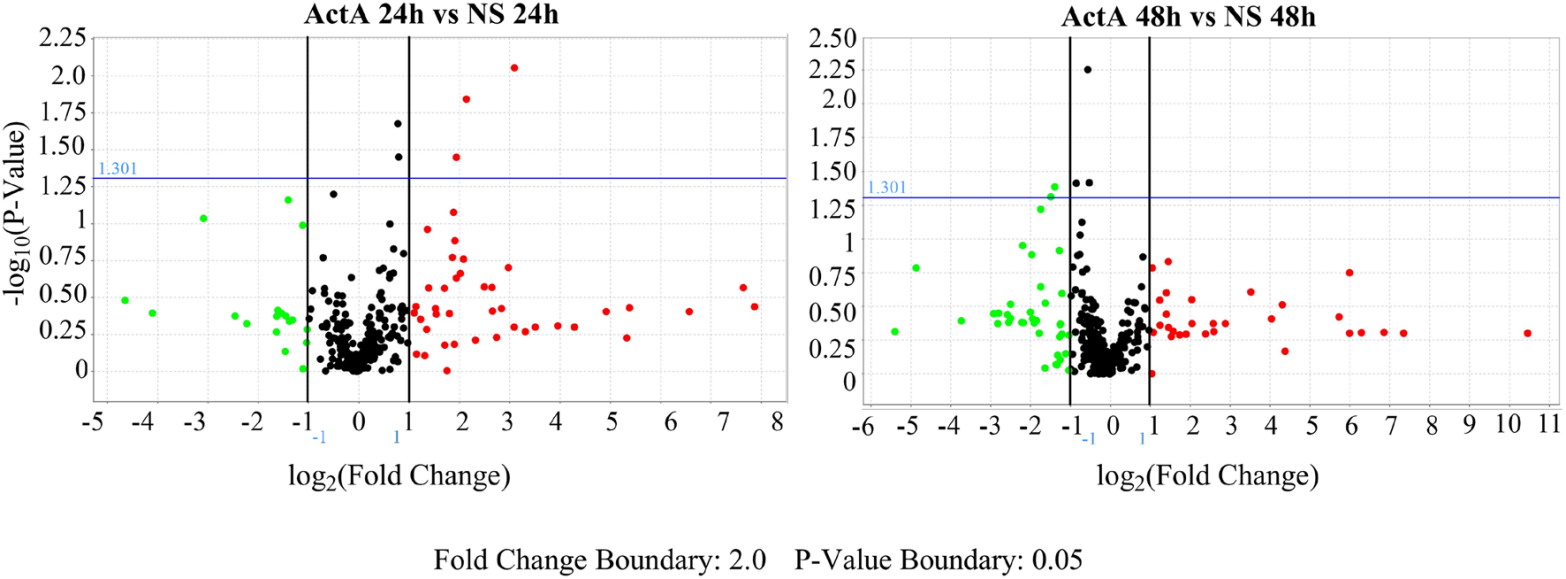
ActivinA-stimulated B-ALL 697 cells secrete EVs enriched in distinctive miRNA species. Volcano plots comparing EV-associated miRNAs from ActivinA-stimulated (EV-ActA) and not stimulated (EV-NS) 697 cells for 24 h and 48 h. The X-axis represents the log2 transformed fold change of each miRNA expression between ActivinA-derived and non-stimulated samples. The Y-axis shows the −log10 of the p-value. Different colored dots show the differential regulation of miRNAs: in red the up-regulated and in green the down-regulated.

To understand the potential mechanisms by which B-ALL cell-derived EVs may mediate cell survival, we investigated the miRNA cargo of EVs derived from 697 cells stimulated (EV-ActA) or not (EV-NS) with ActivinA. OpenArray technology was used to screen EV-miRNA expression among 12 samples. After data cleaning (AmpScore > 1.24), we identified 10 miRNAs that were significantly altered in response to ActivinA stimulation when levels were normalized by three endogenous control genes: RNU44, RNU48 and U6 (Fig. 5 and Table 1). To validate the expression of the top 10 candidate miRNA species obtained at 24 h and 48 h after analysis based on endogenous control genes, we analyzed 20 validation samples, using an OpenArray custom panel. Validation analyses confirmed only two miRNAs that were found as deregulated by ActivinA in the screening array. In particular, miR-491-5p was significantly up-regulated at 24 h in EVs derived from ActivinA-stimulated 697 cells, compared to controls (RQ = 2.92, *p* value < 0.0001) (Table 1). On the contrary, 48 h-ActivinA stimulation significantly decreased the expression level of EV-associated miR-1236-3p (RQ = 0.14, *p* value = 0.0461) (Table 1). In addition, among the other small RNA species, let-7i-3p and miR-18a-3p maintained the same trend of expression observed in the screening phase, without however reaching statistical significance. The overexpression of miR-491-5p in EV-ActA compared to EV-NS was further demonstrated in the Nalm6 cell line, by means of digital PCR (Supplementary Fig. 6). Overall, our data suggest that ActivinA besides stimulating vesiculation, can also influence EV miRNA cargo of B-ALL cells.

**Table 1:** MiRNAs regulated by ActivinA treatment.

miRNA	Treatment	RQ geometric mean	95% CI		Fold change	*P* value
24 h						
let-7i-3p*	ActivinA	4.0E−06	4.7E−07	4.3E−05	1.28	0.82
	Control	4.0E−06	1.6E−07	7.7E−05		
miR-639*	ActivinA	6.0E−06	1.0E−06	4.1E−05	0.29	0.09
	Control	2.2E−05	6.8E−06	7.2E−05		
mir-135b-3p*	ActivinA	3.2E−08	1.7E−08	6.1E−08	0.43	0.21
	Control	7.5E−08	1.8E−08	3.2E−07		
miR-491-5p*	ActivinA	2.5E−04	1.6E−04	4.0E−04	2.92	< 0.0001
	Control	8.7E−05	4.7E−05	1.6E−04		
miR-139-5p*	ActivinA	8.0E−06	1.7E−06	3.3E−05	0.18	0.02
	Control	4.2E−05	2.8E−05	6.5E−05		
48 h						
miR-15b-3p*	ActivinA	0.01	0.003	0.02	1.60	0.47
	Control	0.01	0.002	0.01		
miR-18a-3p*	ActivinA	0.001	4.7E−05	0.02	0.61	0.67
	Control	0.001	2.5E−04	0.01		
let-7e-5p*	ActivinA	2.28	1.28	3.29	2.34	0.01
	Control	0.98	0.67	1.29		
miR-1236-3p*	ActivinA	1.6E−06	3.2E−07	7.6E−06	0.14	0.05
	Control	1.1E−05	9.2E−07	1.4E−04		
miR-23b-3p*	ActivinA	0.002	5.0E−05	0.09	1.28	0.83
	Control	0.002	1.0E−04	0.03		

The table shows the differentially expressed miRNAs validated by RT-qPCR assay in triplicate. The results are presented as the geometric mean and 95% confidence interval (CI) of miRNA expression in EV-ActA relative to EV-NS.

## Discussion

In the last few years, EVs have received considerable attention as important mediators of cell-to-cell communication. Increasing evidence has demonstrated a bidirectional EV-mediated crosstalk between leukemic cells and many other cell types residing within the BM niche. On the one hand, EVs released by leukemic cells have been described to induce angiogenesis^15,18^, inhibit healthy hematopoiesis^19^, modulate immune cells^20,21^ and promote BM niche remodeling, for example by inducing lipolysis, to obtain growth advantage^18,22, 23^*.* On the other hand, it has been found that the microenvironment can support the growth, survival and chemoresistance of leukemic cells through vesiculation^14,24^. In this regard, for instance, MSC-derived EVs are able to promote drug resistance in ALL cells by activating NF-κB signaling axis and to protect CLL cells from apoptosis by increasing the expression of anti-apoptotic molecules^14,25^. Furthermore, EVs have been suggested to contribute to inter-leukemic cell communication. For example, exosomes released by Chronic Myeloid Leukemia (CML) cells have been shown to promote the proliferation and survival of leukemic cells, both in vitro and in vivo, by activating anti-apoptotic pathways^26^*.* This phenomenon has been confirmed also in Acute myeloid leukemia (AML), in which EVs from resistant HL60 can interact with sensitive HL60 cells and transfer their chemo-resistance by overexpressing multidrug resistance protein 1^13^*.* In the context of B-ALL disease, the conveyance of information mediated by EVs among leukemic cells is still poorly investigated. In 2017, Johnson and colleagues demonstrated that B-ALL cells are capable of secreting lEV which were internalized by other leukemic cells leading to phenotypic transformation towards the cell of origin^27^. Moreover, exosomes isolated from the serum of ALL patients or from B-ALL cell conditioned medium were shown to promote leukemic cell proliferation by regulating the ratio between pro-apoptotic and pro-survival genes^17^. We have recently published that ActivinA, a member of the TGF-β family, modulates several genes associated to Ca^2+^ homeostasis, increases intracellular calcium levels and promotes actin polymerization in B-ALL cells^4^. All these biological processes guide cytoskeleton reorganization, which is a crucial event for EV production, secretion and internalization^28,29^. Based on the peculiar processes modulated by ActivinA in leukemic cells, we investigated the effect of this molecule on the so far underexplored vesiculation of B-ALL cells. Interestingly, we highlighted that ActivinA increases the number of total EVs produced by two B-ALL cell lines, namely 697 and Nalm6 cells. ActivinA activity did not impact on a specific EV subpopulation, as both sEV and lEV release were stimulated. Indeed, we can hypothesize that ActivinA promotion of B-ALL vesiculation could be ascribed to its ability to modulate calcium concentration and cytoskeleton remodeling. To support our hypothesis, it has been demonstrated that the secretion of exosomes, which are one of the main sEV subsets, is mediated by increasing levels of intracellular Ca^2+^^30^ and that the formation of lEV, such as microvesicles, requires the activation of Ca^2+^-dependent enzymes and the reorganization of actin cytoskeleton^31^. Furthermore, in line with previous reports demonstrating that B-ALL cells can dialogue to each other by releasing EVs^27^, we also observed that B-ALL cells can actively uptake EVs released by other B-ALL cells. Importantly, B-ALL cells internalized more EVs when cocultured with EV-ActA compared to EV-NS, suggesting once more that, upon ActivinA stimulation, B-ALL cells increase vesiculation, boosting the crosstalk with neighboring leukemic cells. Concerning the possible biological role of this phenomenon, there are different studies reporting that cancer-derived EVs can promote the survival of nearby cancer cells^32,33^. During tumor growth, cancer cells are subjected to a profoundly stressful environment with scarce nutrients and oxygen. In this context, it has been shown that EVs can protect tumor cells from nutrient deprivation-induced cell death^34,35^*.* Accordingly, we aimed at investigating whether ActivinA-mediated enhancement of B-ALL crosstalk through EV release may sustain cell survival in a stressful condition also in the context of B-ALL. Hence, we performed a series of experiments in which 697 cells were cultured for twelve days without changing the culture medium to mimic nutrient starvation, which is a stress condition typical of a highly infiltrated leukemic BM niche. Surprisingly, for the first time, we demonstrated that the addition of EVs to the culture was able to promote leukemic cell survival in a dose-dependent manner. In detail, the pro-survival effect was not observed in the first days of culture, when the leukemic cells were in the log phase, thus suggesting no effect on cell growth, but after reaching the plateau, when a great percentage of untreated 697 cells started to die. It has been already demonstrated that tumor cell-derived EVs enable the transfer of cancer-associated molecules, which are able to alter the phenotype of recipient cells^34,36^. EVs contain and deliver to recipient cells a wide range of bioactive molecules including nucleic acids, anti-apoptotic factors, metabolites and nutrients^37,38^. Indeed, we can postulate that EVs were able to protect B-ALL from stress-induced apoptosis and necrosis, possibly by transferring those bioactive molecules such as nutrients, anti-apoptotic factors or survival-inducing miRNAs, as already demonstrated in the context of other pathologies^14,32^. Since ActivinA has been demonstrated to influence the growth and survival of solid cancer cells, with different outcomes depending on the type^5,39, 40^, we explored the possibility that it could play a role also in the case of B-ALL, in our experimental setting. Of note, we observed that the stimulation of 697 cells with ActivinA was able to protect them from apoptosis under nutritional stress conditions, but it did not influence the growth kinetic in the log phase. Furthermore, we demonstrated that EVs isolated from ActivinA-stimulated cells (EV-ActA) were more effective than EV-NS in inducing protection. Based on our observations that ActivinA boosts B-ALL vesiculation and that B-ALL-derived EVs promote survival in a dose-dependent manner, we can hypothesize that the increased number of EV-ActA could be responsible of the superior viability of EV-ActA-treated B-ALL cells. To understand whether, besides the increased EV quantity, changes in terms of EV cargo could play a role in the phenomenon, we firstly focused on EV-associated miRNAs. A wide range of miRNAs has been described so far to take part to the EV cargo^41^. Moreover, in the context of acute leukemia, it has been previously demonstrated that AML cells are able to transfer miR-155 to another AML cell via EVs^42^. Therefore, we decided to explore whether, besides impacting on EV production, ActivinA could also modulate the EV miRNA cargo of 697 cells. Interestingly, we found two differentially expressed miRNAs in EV-ActA: miR-491-5p, that resulted significantly up-regulated at 24 h and miR-1236-3p, whose expression was significantly decreased at 48 h. It is already known that ActivinA regulates miRNA expression in solid cancer cells^39^, but we discovered that this cytokine can also modulate the level of diverse miRNA species within secreted EVs. Focusing on miR-491-5p, it was demonstrated to play a crucial role in several cancers. It's important to note that microRNAs, including miR-491-5p, can exhibit diverse functional roles, acting as both oncogenes and tumor suppressors depending on the specific cancer type and the molecular pathways they influence^43,44^. Indeed, in breast cancer miR-491-5p was shown to act as tumor suppressor^44^, while in colon cancer it was found upregulated and its expression was correlated with poor prognosis^43^. MiR-491-5p has been demonstrated both in vitro and in vivo to exert a pro-tumoral role also in glioblastoma, by reducing the sensitivity of glioblastoma cells to ferroptosis through the inhibition of the p53/p21 signaling pathway^45^. It was also found increased in cisplatin resistant gastric cancer cells, and its inhibition reverted the therapy sensitivity, inducing cell apoptosis^46^. Moreover, a previous report showed that exosome-associated miRNAs can be shuttled between B-ALL cells regulating cell survival^17^. Further investigations are needed to understand whether miR-491-5p, transported into B-ALL recipient cells by EVs, could take part to the anti-apoptotic effect mediated by EVs, especially in presence of ActivinA. Concerning miR-1236-3p, it has been previously reported that it is able to inhibit proliferation, invasion, migration in several tumor types^47,48^. Accordingly, its onco-suppressive role was demonstrated in vivo in colon cancer and osteosarcoma models^47,49^. Since our published data demonstrated that ActivinA is a leukemia-promoting factor conferring migratory advantage to B-ALL cells^4^, it will be worth investigating whether the downregulation of miR-1236-3p induced by ActivinA could take part to the modulation of B-ALL migratory ability. In line with recent literature^50–52^, our preliminary data indicate that, besides miRNAs, 697-derived vesicles can carry, as a part of their cargo, mRNAs, including the E2A-PBX1 fusion transcript (data not shown) and that ActivinA stimulation could play a role also on the mRNA cargo composition. Future studies are needed to complete the definition of B-ALL-EV cargo and to understand the possible biological role of different molecular species including mRNAs, proteins and lipids on target cells.

In this study we demonstrated that EVs play a crucial role in B-ALL, as having a precise crosstalk with other bystander leukemic cells is vital for the survival of malignant cells. ActivinA turned out to be an interesting cytokine regulating B-ALL cell vesiculation in terms of quantity and miRNA cargo. In accordance with our previous and newly obtained results on the pro-leukemic role of ActivinA^4^, we may hypothesize that within the leukemic BM niche, leukemic cells could stimulate MSCs and other microenvironment elements to produce ActivinA. In turn, the secreted ActivinA could act on leukemic cells that express the ActivinA receptors^4^, resulting in the activation of signaling pathways that control diverse biological processes, including the calcium homeostasis and the cytoskeleton reorganization, which converge in increasing B-ALL cell vesiculation. Moreover, it is conceivable to think that released EVs could be internalized by other recipient B-ALL cells not directly interacting with the BM microenvironment, in order to transfer pro-survival signals.

Overall, the present work, in synergy with data already published by our group^4^, clearly demonstrates that ActivinA can promote leukemia through different mechanisms, including the enhancement of leukemic cell migratory and invasive properties and the increase of EV-mediated B-ALL cell crosstalk and survival. Given its pleiotropic actions, we believe that for potential clinical translation, the optimal approach to counteract the observed pro-leukemic effects, would be to sequester ActivinA, rather than targeting specific deregulated molecular pathways. Indeed, ActivinA blocking by specific molecular traps would be a valuable option to modulate the crosstalk between leukemic cells and potentially making B-ALL cells more sensitive to apoptosis induced by metabolic stress-inducing chemotherapics. At this regard, a few ActivinA molecular traps have been already tested in preclinical models and in clinical protocols in the context of hematological and not hematological diseases^53,54^. Future studies in xenograft B-ALL mouse models are needed to evaluate the potential anti-leukemic action of ActivinA blockers alone or in combination with chemotherapy.

## Material and methods

### Culture of B-ALL cell lines

The B-ALL cell line Nalm6 was purchased from American Type Culture Collection (ATCC, USA), 697 and SUPB-15 cells were purchased from DSMZ (Germany). Cells were cultured in RPMI 1640 medium (Roswell Park Memorial Institute) supplemented with 10% heat-inactivated Fetal Bovine Serum (FBS) (GE Healthcare), penicillin (100 U/mL), streptomycin (100 µg/mL) and l-glutamine (2 mM) (Euroclone, Milan, Italy). 697 and Nalm6 cell lines biological identity was analyzed by cell surface phenotyping (flow cytometry, FACS Canto II, BD Bioscience, San Jose, CA, USA). The 697 cell line was identified as CD3^−^/CD80^−^/CD34^−^/CD13^−^/CD10^+^/CD19^+^/CD38^+^/MHC-II^+^, whereas the Nalm6 cell line was recognized as CD3^-^/CD37^-^/CD80^-^/CD10^+^/CD19^+^/CD138^+^/MHC-II^+^ (anti-human CD3, CD34, CD10, CD19: BD Biosciences; anti-human CD38: eBioscience, San Diego, CA, USA; anti-human MHC-II: BD Pharmingen, San Diego, CA, USA; anti-human CD80, CD37, CD13: ImmunoTools, Germany). Flow cytometry data were analyzed by using FlowJo Software (Tree Star, Inc. Ashland, OR, USA). Cell-specific translocations t(1;19) for 697 cells and t(9:22) for SUPB-15 cells were evaluated by PCR with specific primers (listed below in Table 2) and gel electrophoresis. Concerning the Nalm6 cells, their identity was confirmed by FISH assay using XL 5q32 PDGFRB BA Probes (MetaSystems, GmbH), acquisition and analysis by Neon 1.2.9 software (MetaSystems, GmbH).

**Table 2:** Specific primers for cell-specific translocations t(1;19) and t(9;22).

Forward t(1;19)	CACCAGCCTCATGCACAAC
Reverse t(1;19)	TCGCAGGAGATTCATCACG
Forward t(9;22)	GACTGCAGCTCCAATGAGAAC
Reverse t(9;22)	GTTTGGGCTTCACACCATTCC

### B-ALL cell line stimulation for EVs production

697 and Nalm6 cell lines were maintained in RPMI 1640 complete medium supplemented with 10% FBS, washed two times in saline solution and switched to serum-free medium to avoid contaminations by FBS-derived vesicles. The cells were seeded at a concentration of 2 × 10^6^ cell/ml, in order to favor vesiculation and therefore cell to cell communication. Then, the cells were treated or not (NS) with recombinant Human/Mouse/Rat ActivinA (R&D Systems) at 50 ng/ml or 200 ng/ml, depending on the experiment for 24 and/or 48 h, depending on the experiment.

### Isolation and quantification of EVs from 697 and Nalm6 cells treated or not with ActivinA

To investigate the quantity and the size of EVs released by B-ALL cells, we performed Nanoparticle tracking analysis (NTA) (NanoSight NS300 System, Malvern Panalytical) on culture supernatants. Five recordings of 30’’ each, were made for all samples. Collected data were analyzed with NTA software (3.2 and 3.3 versions). Based on their size, EVs were categorized into small extracellular vesicles (sEV) (30–150 nm) and large extracellular vesicles (lEV) (151–750 nm).

To isolate EVs released by B-ALL cells, cell culture supernatants were firstly centrifuged at 1000, 2000, and 3000×*g* for 15 min at 4 °C and then processed by ultracentrifugation at 110,000×*g* for 97 min at 4 °C (Ultracentrifuge Beckman L90K with the rotor 50.2 Ti) The isolated EVs were suspended in PBS.

### Characterization of EVs: flow cytometry, western blot

The immunophenotype of EVs was evaluated by flow cytometry (MACSQuant, Miltenyi Biotec). To analyze EV integrity, 60 µl aliquots of ultracentrifuged samples were stained with 0.02 µM CFSE (5(6)‐carboxyfluorescein diacetate N‐succinimidyl ester) at 37 °C for 20 min (Thermo Fisher Scientific). Each aliquot of CFSE‐stained EVs was incubated with CD19-APC, CD9-APC and CD63-APC antibodies (Miltenyi Biotec). Gating strategy is illustrated in Supplementary Fig. 7. Before use, each antibody was centrifuged at 17,000×*g* for 30 min at 4 °C to eliminate aggregates. A stained PBS control sample was used for background normalization. The analyses were performed by means of MACS-Quantity (Miltenyi Biotec). For the western blot evaluation of EVs markers, cells and isolated EVs were lysed with RIPA lysis buffer containing (1% of NP-40, 0.5% Na-Deoxyxholate, 350 mM NaCl, 0.1% SDS and supplemented of 1% of Protease Inhibitor Cocktail and 0.25 mM of Phenyl-methyl-sulfonyl fluoride, all from SigmaAldrich). The lysis was performed on ice for 30 min, then the samples were centrifuged at 4 °C for 15 min at 21,000×*g* to remove cell debris. The supernatant was collected in a new tube and stored at −20 °C until usage. Proteins concentration of all samples was determined by Bicinchoninic Acid Assay (BCA-Thermo-Scientific™ Pierce™) and the absorbance was detected by using a spectrophotometer (Magellan, Tecan Life Science). Protein electrophoresis was performed by loading 10 µg of proteins in NuPAGE™ 4–12% Bis–Tris Gel (ThermoFisher). We used non-reducing condition for evaluation of CD81 and reducing condition for CD9. Then, we proceeded to transfer the proteins to a PVDF membrane by means of IBlot 2 Gel Transfer Device (ThermoFisher). The primary antibodies used were the monoclonal mouse anti-human CD81 (1:1000, Invitrogen), the monoclonal rabbit anti-human CD9 (1:1000, Abcam) and the monoclonal mouse anti-human β-Actin (1:6000, SigmaAldrich). The secondary antibodies used were the anti-mouse IgG (Fc specific)-Peroxidase (1:20,000, SigmaAldrich) and the anti-rabbit IgG (Fc specific)-Peroxidase (1:10,000, Prodotti Gianni Srl). To evaluate protein expression, we performed enhanced chemiluminescent assay utilizing the Westar Antares chemiluminescent substrates (Cyanagen); image acquisition was performed by using Alliance Q9 Advanced imaging system (Uvitec).

### Analysis of EVs uptake by B-ALL cell lines: flow cytometry and confocal imaging

EVs were isolated from 40 × 10^6^ 697 or Nalm6 cells stimulated or not with ActivinA (200 ng/ml), as previously described, resuspended in 60 µl of PBS and labeled or not with CFSE 0.2 mM, as described above. After 24 h, EVs were washed twice with PBS by ultracentrifugation at 110,000×*g* for 97 min at 4 °C, in order to pellet EVs and remove the excess of dye. EVs labeled or not with CFSE were directly added for 24 h to 0.5 × 10^6^ 697 or Nalm6 cells cultured in RPMI 1640 medium without FBS but supplemented with penicillin (100 U/mL), streptomycin (100 µg/mL) and l-glutamine (2 mM) under standard culture conditions (37 °C, 5% CO_2_). EV uptake was assessed by evaluating CFSE mean fluorescence intensity (MFI) in B-ALL cells cocultured with stained or unstained EVs, by means of Flow Cytometry (FACS Canto II, BD Bioscience, San Jose, CA, USA). 697 and Nalm6 cells, labeled with 1 µM CFSE (5 × 10^6^ cells for 10 min in 1 ml of PBS at room temperature in the dark, then + 5 ml of RPMI 1640 10% FBS for 5 min on ice) and then washed with PBS, were used as positive fluorescence controls. Unlabeled cells were instead used as negative control. Data were analyzed by FlowJo Software (Tree Star, Inc. Ashland, OR, USA). EV uptake by 697 and Nalm6 cell lines was further investigated by confocal microscope imaging. At this purpose, 50,000 cells cocultured with CFSE-labeled or unstained EVs were spotted on slides pre-coated with 100 µg/ml of polysine (SigmaAldrich) to promote adhesion for 15 min at 37 °C in the incubator into a humidified cassette. After washing with PBS, the slides were fixed with 4% PFA (Paraformaldehyde, Sigma Aldrich) for 20 min at room temperature and then washed three times with PBS. To stain cell nuclei, we performed DAPI staining (1000×, Cell biolabs, Inc.) for 15 min at room temperature in the dark. In order to preserve the slides and avoid drying, we added one drop of aqueous mounting medium (SigmaAldrich) and covered the slides with a coverslip. Cellular uptake of EVs was evaluated by using a confocal laser-scanning microscope (Zeiss LSM 710 with Airyscan) with a 63×, 1.4 N/A oil-immersion objective acquiring the entire cell volume with a z-stack protocol. Laser intensities and acquisition parameters were held constant throughout each experiment.

Confocal microscopy fields were analyzed using specific homemade-designed macro with ImageJ (https://imagej.nih.gov/ij/) software. In detail CFSE signal intensity was analyzed measuring the ID and normalized over the signal deriving from unlabeled cells. All the data obtained derived from at least 10 fields per experimental condition.

### Cell growth assay

To investigate the effect of EVs on B-ALL cell growth and survival, 697 cells were seeded in a 24 multiwell plate at 0.5 × 10^6^ cell/ml in complete RPMI 1640 medium. Three replicates were performed for each condition. Starting from day 0 of culture, EVs were freshly isolated from 25 × 10^6^ or 80 × 10^6^ of 697 cells stimulated or not for 24 h with ActivinA (200 ng/ml), resuspended in 60 µl of PBS and then added every 72 h to the three wells (20 µl/well) for three times. On the same days, 20 µl of PBS or ActivinA (50 ng/ml) were added to three different wells of cultured 697 cells. The medium of the culture was never changed until the end of the experiment. Overall, four experimental groups were adopted: (1) PBS-stimulated 697 cells; (2) ActivinA-stimulated 697 cells; (3) 697 cells stimulated with EVs isolated from other unstimulated 697 cells; (4) 697 cells stimulated with EVs isolated from other ActivinA-stimulated 697 cells. The number of viable cells were evaluated from day 0 up to day 12, by trypan blue staining and cell count by means of an automated cell counter (Countess II, Invitrogen).

### Evaluation of EV-associated miRNAs: screening and validation

697 cells were cultured at concentration of 2 × 10^6^ cell/ml in RPMI 1640 without FBS and then stimulated or not with ActivinA (50 ng/ml) for 24 h or 48 h. EVs were purified as previously described and EV-associated miRNAs were isolated from frozen 697-derived EVs pellets by using the miRNeasy and Rneasy CleanUp Kit (Qiagen), according to the manufacturer’s instructions. The total yield and the purity were estimated by 2100 Bioanalyzer (Agilent Technologies, Santa Clara, CA) using Agilent RNA 6000 Pico Kit. The final purified miRNA-enriched RNA was eluted into 20 µl of RNAse-free water and stored at −80 °C until further use.

For the screening phase, miRNAs were prepared by standard reverse transcription (RT) from 40 ng and pre-amplification procedures, followed by RT-qPCR analysis with the QuantStudio 12 K Flex OpenArray Platform (QS12KFlex, Applied Biosystems). Three biologically independent experiments were tested for each condition, stimulated or not stimulated with ActivinA for 24 h and 48 h. In detail, RT was performed by using Megaplex™ RT Primers, Pool A v2.1 and Pool B v3.0, with the TaqMan^®^ Micro RNA Reverse Transcriptase Kit (Life Technologies, Foster City, CA). Each cDNA was pre-amplified in 14 cycles, diluted 1:20 with nuclease-free water and stored at 4 °C until expression analysis with the OpenArray^®^ System. The reaction mix was analyzed with the QuantStudio™ 12K Flex Real-Time PCR System with the OpenArray^®^ Platform (QS12KFlex, Applied Biosystems), according to the manufacturer’s instructions. In particular, we obtained 758 Ct (Threshold Cycle) values for each condition (n = 12), which included 755 unique miRNAs and three internal controls (RNU48, RNU44 and U6)^55^.

The top-5 miRNAs differentially expressed after ActivinA exposure in each time-point were validated in 384-Plates in triplicate with the QuantStudio™ 12K Flex Real-Time PCR System (n = 8). RT was performed by using the Custom Primers Pool and TaqMan^®^ MicroRNA Reverse Transcriptase Kit (Life Technologies). Pre-amplification and analyzing conditions were the same as the screening phase.

### Digital PCR

10 ng of total RNA were reverse-transcribed using Mircury LNA RT kit (Qiagen) according to manufacturer’s instructions. miRNA digital droplet PCR (ddPCR) was performed using EvaGreen supermix (Bio-Rad) and the following miRCURY LNA PCR primer sets (Exiqon): hsa-miR-491-5p (ID 204695), hsa-miR-16-5p (ID 205702), SNORD48 (ID 203903), UniSP6 (ID 203956). Each ddPCR assay mix (22μL) containing 11microL of EvaGreen master mix, 1,1 of miRCURY LNA (20×) primer mix and 50 to 80 pg of cDNA was loaded into a disposable droplet generator cartridge for the automated Bio-Rad Droplet Generator machine. After droplet generation, PCR plates were thermally sealed and PCR was initiated immediately. Thermal cycling conditions for EvaGreen assays were as follows: 95 °C for 5 min (ramp rate 2 °C/s), then 40 cycles of 95 °C for 30 s and 56 °C for 1 min and three final steps at 4 °C for 5 min, 90 °C for 5 min, and a 4 °C indefinite hold to enhance dye stabilization. A no template control (NTC) and a negative control for each reverse transcription reaction (RT-neg) were included. Artificial RNA spike-in UniSP6 was added to the assay to monitor RT reaction efficacy and PCR plate loading. We performed each single PCR assay in duplicate. Raw copies/μL for miR-126 were normalized on the geometric mean of two selected housekeeping small RNAs (SNORD48 and miR-16). For cross-condition and cross-experiment comparisons Normalized miR-491-5p copies/RNA pg input were calculated.

### Statistical analysis

Differences between subgroups were compared with the nonparametric tests Wilcoxon matched-pairs two-tailed test, Mann–Whitney two-tailed test or with the parametric tests paired two-tailed t-test and unpaired two-tailed t-test. In case of multiple comparisons we performed the two-way ANOVA with Tukey’s correction. One sample t-test was instead used in case of Fold Change calculation. Statistical differences with a P value of 0.05 or less were considered significant. Data were analyzed and graphics were constructed using GraphPad Prism 5.0 (GraphPad Software, San Diego, CA, USA).

We performed the analysis of the screening data and the normalization of miRNAs through the Gene Expression Suite Software. miRNAs with Ct value < 28 or AmpScore > 1.24 were considered amplified, resulting in 251 miRNAs being included in the analysis. The expression data were normalized using the average Ct of RNU48, RNU44 and U6. Relative quantification of each miRNA was calculated using the RQ = 2^(−∆ct)^ formula, with ^−^∆ct = measured ct− medium_normalization ct^56^.

The differentially expressed miRNAs were identified with fold-change (FC) filtering and *p*-value < 0.05. In the validation phase, for each miRNA, we applied linear regression models with repeated measurements to verify the association between ActivinA exposure and miRNA expression. MiRNA expression values were log2-transformed to achieve a normal distribution. Geometric means and 95% Confidence Interval (CI) of relative quantification were calculated and compared, their ratio (ActivinA exposure/control group) was used to obtain the FC. The statistical analysis was performed using SAS software (version 9.4, SAS Institute, Milan, Italy). A two-sided *p*-value of less than 0.05 was considered statistically significant.

### Supplementary Information

Supplementary Information.

## Data Availability

Microarray data are deposited in the ArrayExpress database (https://www.ebi.ac.uk/arrayexpress), with the accession number E-MTAB-14003.

## Abbreviations

BM: Bone marrow
B-ALL: B-acute lymphoblastic leukaemia
CLL: Chronic lymphocytic leukaemia
CML: Chronic myeloid leukaemia
AML: Acute myeloid leukaemia
EVs: Extracellular vesicles
MSCs: Mesenchymal stromal cells
miRNA: MicroRNA
A.U.: Arbitrary units
MFI: Mean fluorescence intensity
NTA: Nanoparticle tracking analysis
sEV: Small extracellular vesicles
lEV: Large extracellular vesicles
