# Supplementary material for: ActivinA modulates B-acute lymphoblastic leukaemia cell communication and survival by inducing extracellular vesicles production
Source: Sci Rep. 2024 Jul 12;14:16083. doi: 10.1038/s41598-024-66779-3 (PMC11239915; doi:10.1038/s41598-024-66779-3)
Supplement: Supplementary file 1 — Supplementary Information. [file 41598_2024_66779_MOESM1_ESM.pdf]

ActivinA modulates B-Acute Lymphoblastic Leukaemia cell communication and survival by inducing extracellular vesicles production

Eugenia Licari[a], Giulia Cricrì[a,b], Mario Mauri[c], Francesca Raimondo[d], Laura Dioni[e], Chiara Favero[e], Alice Giussani[a], Rita Starace[a], Silvia Nucera[a,c], Andrea Biondi[f,c], Rocco Piazza[c,g], Valentina Bollati[e,h,i], Erica Dander[a#] and Giovanna D'Amico[a#*]

[#]co-last authorship [*]corresponding author

Affiliations:

[a]Tettamanti Center, Fondazione IRCCS San Gerardo dei Tintori, Monza, Italy

[b]Paediatric Nephrology, Dialysis and Transplant Unit, Fondazione Ca' Granda IRCCS Ospedale Maggiore Policlinico, Milano, Italy

[c]Department of Medicine and Surgery, University of Milano-Bicocca, Monza, Italy

[d]Clinical Proteomics and Metabolomic Unit, School of Medicine and Surgery, University of Milano-Bicocca, Monza, Italy

[e]EPIGET Lab, Department of Clinical Sciences and Community Health, University of Milan, Milan, Italy

[f]Pediatrics, Fondazione IRCCS San Gerardo dei Tintori, Monza, Italy

[g]Hematology Division and Bone Marrow Unit, Fondazione IRCCS San Gerardo dei Tintori, Monza, Italy

[h]CRC, Center for Environmental Health, University of Milan, Milan, Italy

[i]Occupational Health Unit, Fondazione IRCCS Ca' Granda-Ospedale Maggiore Policlinico, Milan, Italy

Supplementary Figures

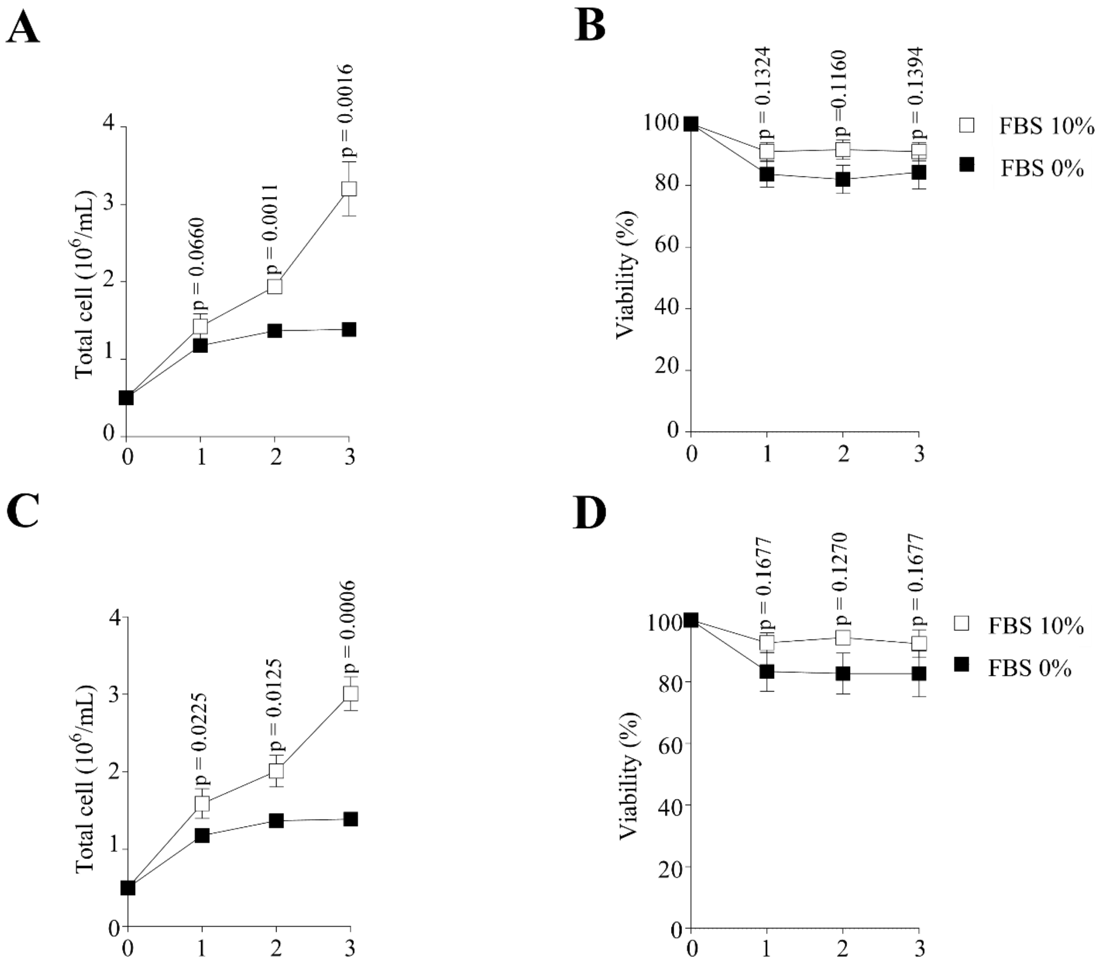

Supplementary Figure 1

Supplementary Figure 1_Serum-deprivation does not impact on 697 and Nalm6 cell viability

697 **(A-B)** and Nalm6 **(C-D)** cells were cultured in complete (10% FBS) or serum-deprived medium (0% FBS) and were kept in culture for 3 days without changing medium. The panels show the growth curves **(A-C)** and the percentage of viable cells **(B-D)** evaluated after trypan blue staining on an automated cell counter. One representative experiment out of two performed is shown. Black square: 0% FBS medium; white square: 10% FBS medium. The data are expressed as mean ± SD (n=3 replicate wells). Unpaired two- tailed t-test.

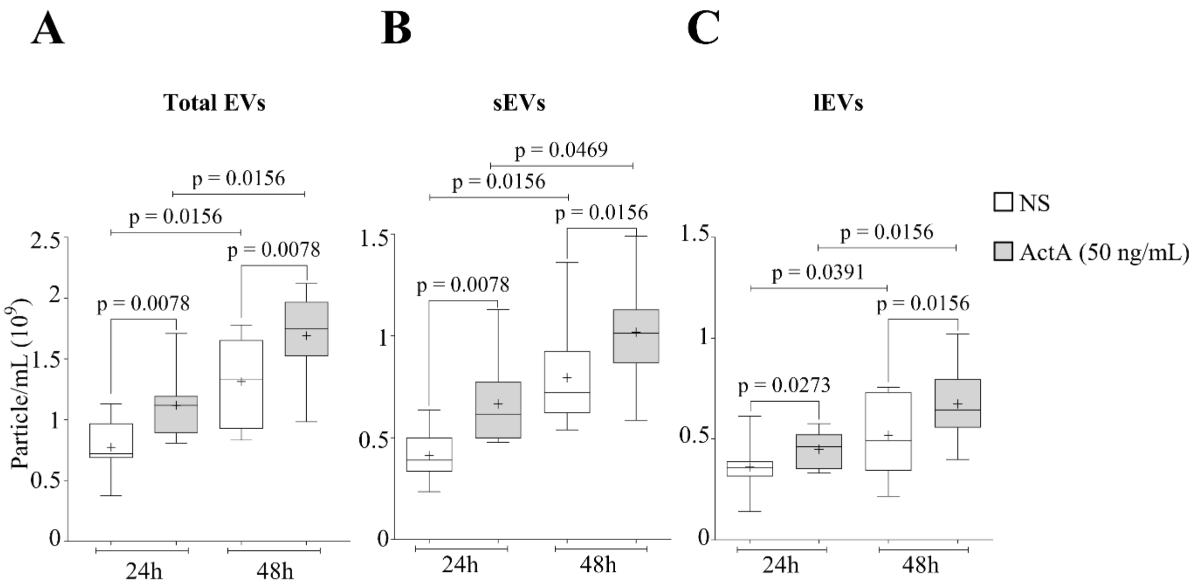

Supplementary Figure 2

Supplementary Figure 2_ActivinA 50 ng/ml promotes 697 cells EVs production

(A-B-C) 697 cells were pretreated or not with ActivinA 50 ng/ml, for 24 and 48 h. The concentration (particle/ml) of EVs in the supernatant was determined by means of NTA. The box plot graphs represent the concentration of total EVs (**A**), sEV (**B**) and lEV (**C**) released by 697 cells. Each box plot shows the median, the mean (+) and extends from the lowest to the highest value (*n*=8 for 697 cells independent experiments per condition). Wilcoxon matched-pairs two-tailed test.

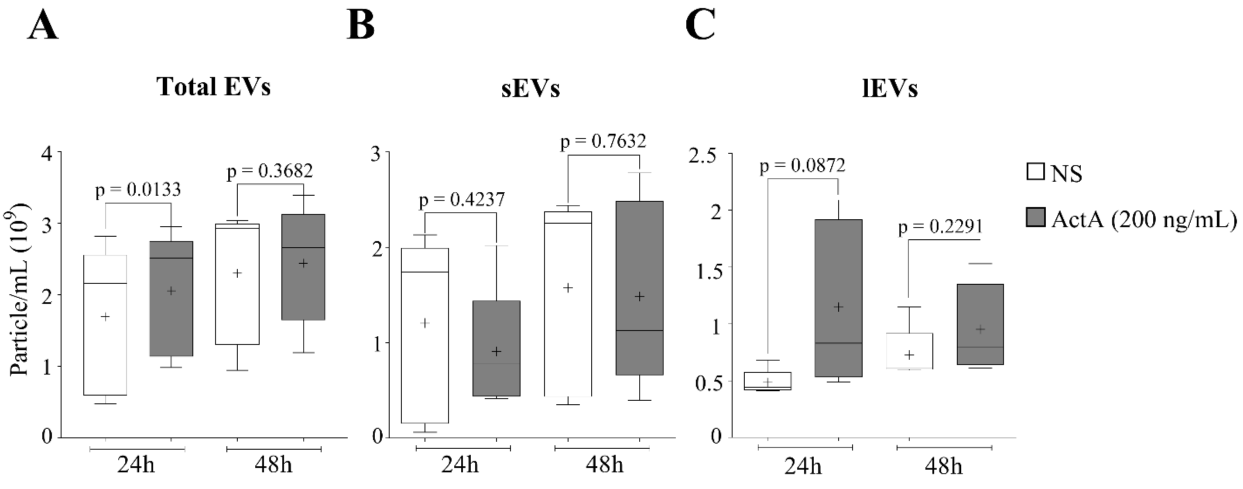

Supplementary Figure 3

Supplementary Figure 3_ ActivinA 200 ng/ml promotes SUPB-15 cells EVs production

(A-B-C) SUPB-15 cells were pretreated or not with ActivinA 200 ng/ml, for 24 and 48 h. The concentration (particle/ml) of EVs in the supernatant was determined by means of NTA. The box plot graphs represent the concentration of total EVs (**A**), sEV (**B**) and lEV (**C**) released by SUPB-15 cells. Each box plot shows the median, the mean (+) and extends from the lowest to the highest value (n=5 independent experiments). Paired two-tailed t test.

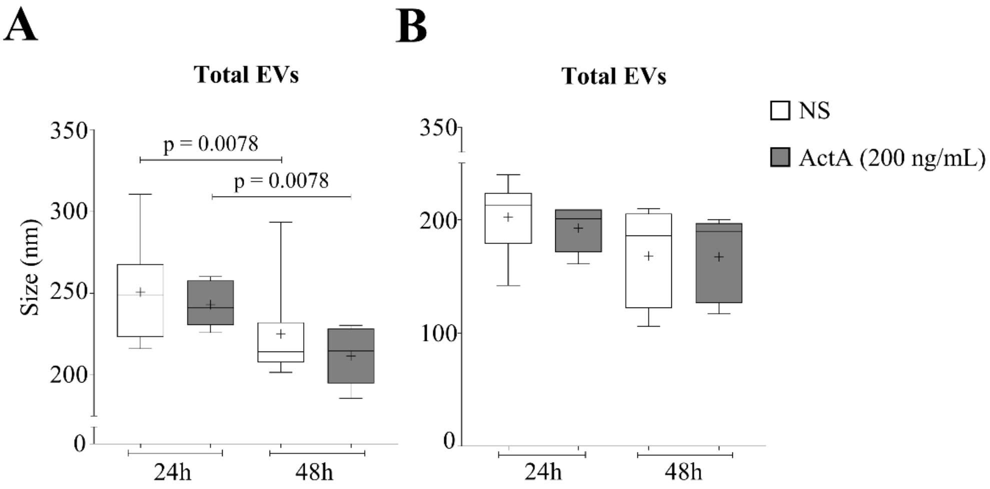

Supplementary Figure 4

Supplementary Figure 4_EVs size distribution

(A-B) 697 and Nalm6 cell lines were stimulated or not with ActivinA 200 ng/ml, for 24 and 48h. The size (nm) of EVs in the supernatant was determined by means of NTA. The box plot graphs represent the size of total EVs purified from 697 **(A)** or Nalm6 cells **(B).** Each box plot shows the median, the mean (+) and extends from the lowest to the highest value (*n*=8 for 697 cells and n=6 for Nalm6 cells independent experiments per condition). Wilcoxon matched-pairs two-tailed test.

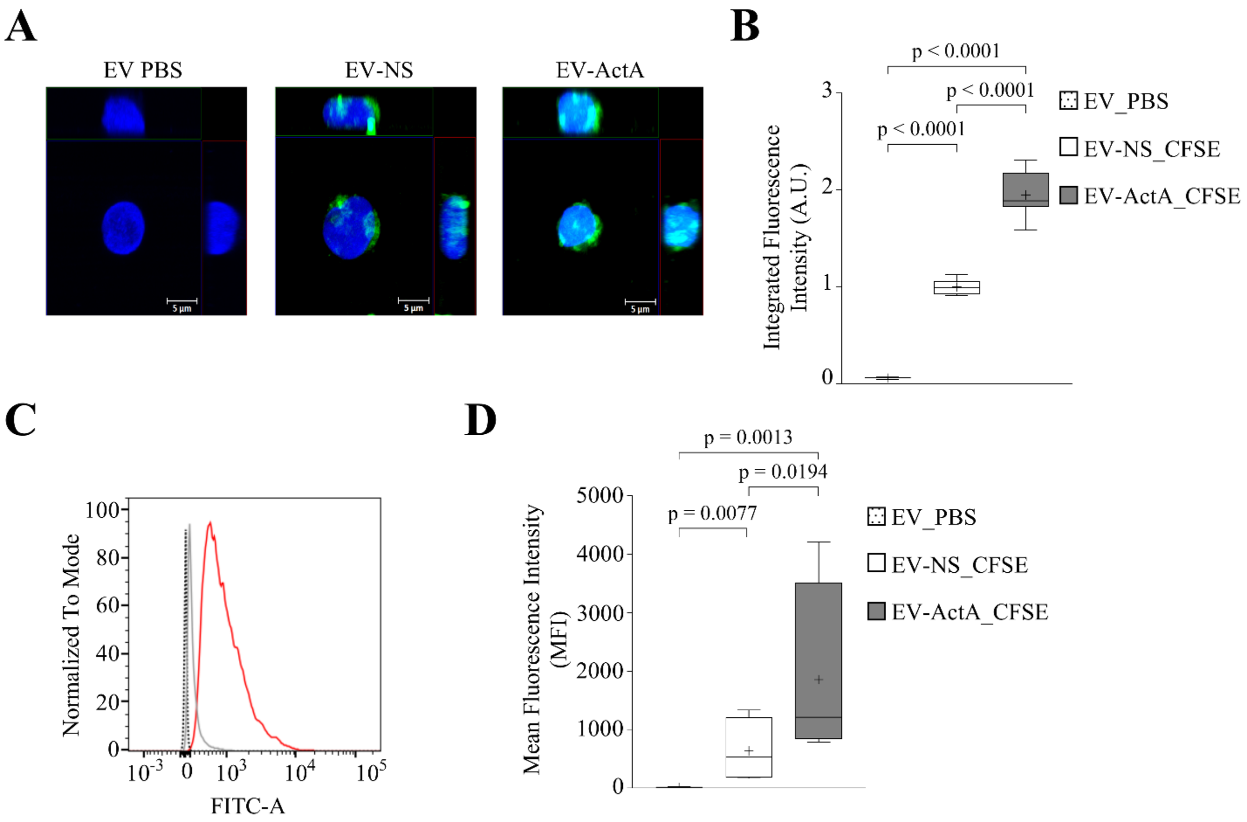

Supplementary Figure 5

Supplementary Figure 5_B-ALL Nalm6 cells can uptake EVs

EVs were isolated from Nalm6 cells stimulated (EV-ActA) or not (EV-NS) with ActivinA (200 ng/ml). EV-NS and EV-ActA were stained with CFSE (green fluorescent dye) or PBS (EV-PBS, negative control) and cocultured with Nalm6 cells for 24h. **(A)** Representative confocal pictures of Nalm6 cells after internalization of EV-PBS, EV-NS and EV-ActA. The green fluorescent dots around the cell nucleus (DAPI staining, blue) indicate internalized EVs. **(B)** Confocal microscopy quantification of CFSE Integrated Fluorescence Intensity. Each box plot shows the median and the mean (+) and extends from the lowest to the highest value (*n*=2 independent experiments). Two-way ANOVA with Tukey's correction for multiple comparisons. **(C)** Representative overlay histogram showing CFSE fluorescence evaluated by flow cytometry in Nalm6 cells cultured with CFSE-stained EV-NS (gray) or EV-ActA (red). Unstained Nalm6 cells were used as negative control (dashed gray line), whereas Nalm6 cells stained with CFSE were utilized as positive control (green). **(D)** Flow cytometry quantification of CFSE MFI in Nalm6 cells cultured with CFSE-labeled EV-NS and EV-ActA. Each box plot shows the median, the mean (+) and extends from the lowest to the highest value (*n*=4 independent experiments). Paired two-tailed t test.

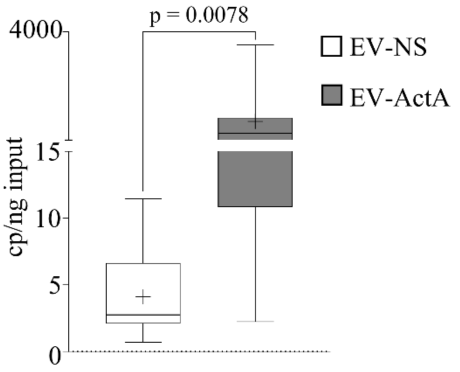

Supplementary Figure 6

Supplementary Figure 6_miR-491-5p is enriched in EV-ActA derived from Nalm6 cell line

MiR-491-5p expression was validated by digital PCR in EVs derived from Nalm6 cells stimulated or not with ActivinA (200 ng/ml) for 24h. Each box plot shows the median, the mean (+) and extends from the lowest to the highest value (*n*=8 independent experiments). Wilcoxon matched-pairs two-tailed tes**t.**

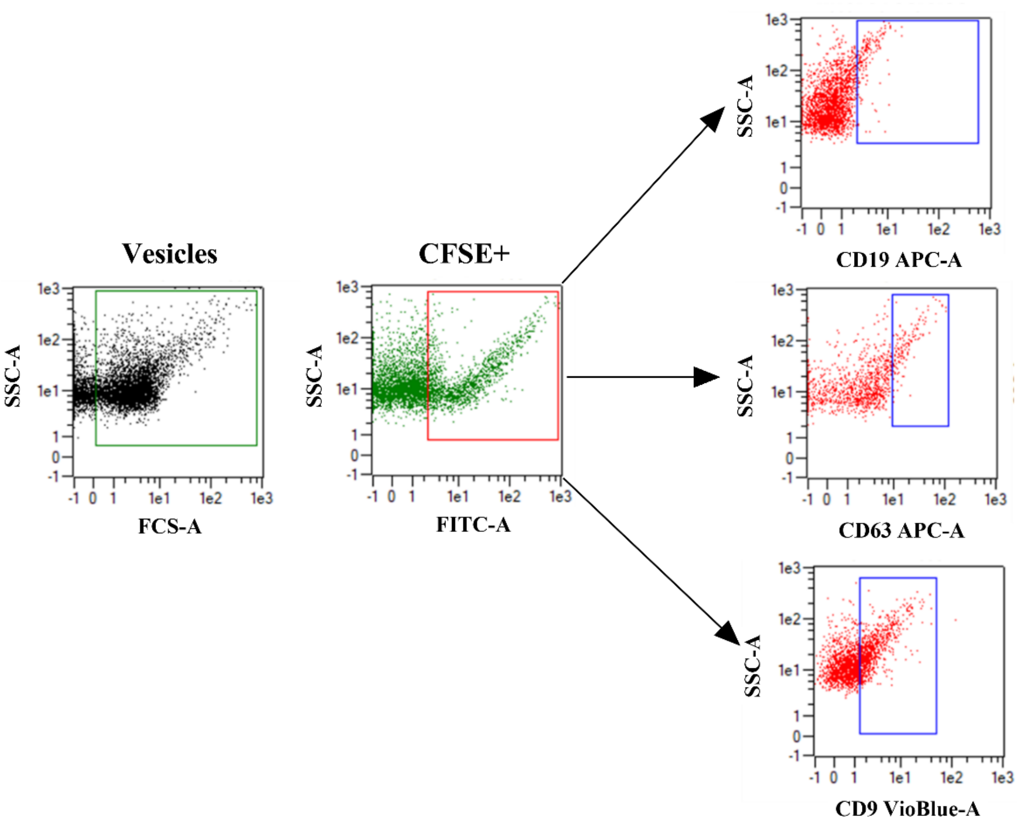

Supplementary Figure 7

Supplementary Figure 7_Characterization of EVs by flow cytometry: gating strategy

EVs purified from 697 cells were visualized, by flow cytometry, as CFSE-positive CD19+ and CD63+ or CD9+ events.

A

Two-way ANOVA with Tukey's correction for multiple comparison

Comparisons	Adjusted P Value	Comparisons	Adjusted P Value
**Day 6**		**Day 9**	
NS *vs* EV-NS_25x10^{6}	0,0003	NS *vs* EV-NS_25x10^{6}	0,0858
NS *vs* EV-NS_80x10^{6}	<0,0001	NS *vs* EV-NS_80x10^{6}	<0,0001
EV-NS_25x10^{6} *vs* EV-NS_80x10^{6}	0,0299	EV-NS_25x10^{6} *vs* EV-NS_80x10^{6}	0,0007
**Day 7**		**Day 10**	
NS *vs* EV-NS_25x10^{6}	0,7029	NS *vs* EV-NS_25x10^{6}	0,7085
NS *vs* EV-NS_80x10^{6}	<0,0001	NS *vs* EV-NS_80x10^{6}	0,0002
EV-NS_25x10^{6} *vs* EV-NS_80x10^{6}	<0,0001	EV-NS_25x10^{6} *vs* EV-NS_80x10^{6}	0,0027
**Day 8**		**Day 12**	
NS *vs* EV-NS_25x10^{6}	0,1838	NS *vs* EV-NS_25x10^{6}	0,7624
NS *vs* EV-NS_80x10^{6}	<0,0001	NS *vs* EV-NS_80x10^{6}	0,4946
EV-NS_25x10^{6} *vs* EV-NS_80x10^{6}	<0,0001	EV-NS_25x10^{6} *vs* EV-NS_80x10^{6}	0,8782

B

Two-way ANOVA with Tukey's correction for multiple comparison

Comparisons	Adjusted P Value	Comparisons	Adjusted P Value
**Day 5**		**Day 8**	
NS *vs* ActA (50 ng/mL)	0,9883	NS *vs* ActA (50 ng/mL)	<0,0001
NS *vs* EV-NS_80x106	0,0052	NS *vs* EV-NS_80x106	<0,0001
NS *vs* EV-ActA_80x106	0,0003	NS *vs* EV-ActA_80x106	<0,0001
EV-NS_80x106 *vs* EV-ActA_80x106	0,8413	EV-NS_80x106 *vs* EV-ActA_80x106	0,0004
**Day 6**		**Day 9**	
NS *vs* ActA (50 ng/mL)	0,0044	NS *vs* ActA (50 ng/mL)	<0,0001
NS *vs* EV-NS_80x106	0,0168	NS *vs* EV-NS_80x106	0,0681
NS *vs* EV-ActA_80x106	<0,0001	NS *vs* EV-ActA_80x106	<0,0001
EV-NS_80x106 *vs* EV-ActA_80x106	0,206	EV-NS_80x106 *vs* EV-ActA_80x106	0,007
**Day 7**		**Day 10**	
NS *vs* ActA (50 ng/mL)	<0,0001	NS *vs* ActA (50 ng/mL)	<0,0001
NS *vs* EV-NS_80x106	0,0003	NS *vs* EV-NS_80x106	0,8812
NS *vs* EV-ActA_80x106	<0,0001	NS *vs* EV-ActA_80x106	<0,0001
EV-NS_80x106 *vs* EV-ActA_80x106	0,0002	EV-NS_80x106 *vs* EV-ActA_80x106	<0,0001
**Day 12**			
NS *vs* ActA (50 ng/mL)	<0,0001		
NS *vs* EV-NS_80x106	0,9701		
NS *vs* EV-ActA_80x106	0,001		
EV-NS_80x106 *vs* EV-ActA_80x106	0,0002		

Supplementary Table 1

Supplementary Table 1_Actual p values of Figure 4

(A) EV-NS were isolated from the supernatant of $25x10^6$ (EV-NS_$25x10^6$) or $80x10^6$ (EV-NS_$80x10^6$) unstimulated 697 cells and added for three times to 697 cells that were kept in culture for 12 days without changing medium. PBS-stimulated 697 cells were used as unstimulated control (NS). The table shows the evaluated comparisons and the specific Adjusted p Value obtained with the statistical test of two-way ANOVA with Tukey's correction for multiple comparison.

(B) 697 cells were stimulated or not with ActivinA 50 ng/ml or with PBS (NS) for three times. EVs were isolated from the supernatant of $80x10^6$ 697 cells unstimulated (EV-NS_$80x10^6$) or stimulated with ActivinA (EV-ActA_$80x10^6$) and added three times to 697 cells that were kept in culture for 12 days without changing medium. The table shows the evaluated comparisons and the specific Adjusted p Value obtained with the statistical test of two-way ANOVA with Tukey's correction for multiple comparison.
